# Singaporean attitudes to cognitive enhancement: a cross-sectional survey

**DOI:** 10.1136/jme-2024-110490

**Published:** 2025-02-25

**Authors:** Casey M Haining, Hui Jin Toh, Julian Savulescu, G Owen Schaefer

**Affiliations:** 1 University of Melbourne, Carlton, Victoria, Australia; 2 Centre for Biomedical Ethics, Yong Loo Lin School of Medicine, National University of Singapore, Singapore; 3 Uehiro Oxford Institute, University of Oxford, Oxford, UK

**Keywords:** Reproductive Medicine, Genetic Enhancement

## Abstract

Recent developments in genetic technologies have provided prospective parents with increasing opportunities to influence their future child’s phenotype. This study aimed to understand public attitudes towards gene-based technologies and services, with a particular focus on improving educational outcomes. We conducted a cross-sectional survey among a Singaporean population (n=1438), adapting a survey instrument previously used in the US context. Our results suggested that Singaporeans had a greater moral acceptance of, and willingness to use, genetic technologies and services compared with the US population. Among the technologies examined, the use of polygenic embryo screening was considered more acceptable than gene editing. While these public attitudes show some support for the use of these technologies, further research and consultation among multiple stakeholder groups is necessary to determine whether such technology should be used and how it should be regulated.

## Introduction

 Increasing access to genetic information has meant that prospective parents have had the opportunity to exercise some control over their future child. To date, selection has primarily been limited to monogenic disorders and traits. Many traits, however, are polygenic, meaning that their expression is the product of a confluence of several genes and environmental factors. Advances in genetic technologies give prospective parents opportunities to influence their future child’s phenotype.

One possible means by which this can be achieved is through polygenic embryo screening (PES),^i^ whereby prospective parents can select an embryo during preimplantation genetic testing as part of the in vitro fertilisation (IVF) process based on calculated polygenic risk scores (PRS). While PES is not widely available and contested,^1^,^6^ there is evidence that PES has been used in China^7^ and is being offered commercially by a small number of companies in the USA.^8 9^ While many commercial companies limit the use of PES to medical traits, it was recently reported that a US startup, Heliospect, was preparing to launch a service that would allow parents to select for non-medical traits such as IQ and height.^10^ In Singapore, the country which this article is concerned with, PES is currently not permitted under existing regulations.^11^

Parents may also be able to exert some control over their future child’s phenotype through the use of germline genome editing such as through clustered regularly interspaced short palindromic repeats (CRISPR) technology, which could be used to make edits to gametes or embryos to influence the future child’s phenotype (hereafter referred to as gene editing).^12^ The use of gene editing remains restricted and is not permitted for therapeutic (or clinical purposes) in Singapore.^13 14^ ^ii^

While these two genetic technologies bring many opportunities, their use remains contested. Studies assessing public attitudes towards such technology have demonstrated some support for their clinical use; however, such support is contingent on their intended use. Indeed, greater support is shown for using these technologies for the purposes of improving health and preventing disease, whereas when such technologies are used for non-medical purposes they garner less support.^15 16^ In addition, such views are likely to be heavily influenced by cultural values. While much of the attention to date has been given to ascertaining views among Western cohorts, this study aims to capture the attitudes of Singaporeans. Singaporeans represent a cross-section of Asian populations (74.3% Chinese, 13.5% Malay, 9% Indians and 3.2% others). In this paper, we report on the results of a survey carried out with a Singaporean population that explored participants’ views about the acceptability and willingness to use different gene technologies in the context of improving educational outcomes. We report this study in accordance with the Consensus-Based Checklist for Reporting of Survey Studies (CROSS) guidelines.^17^

## Methodology

### Survey development and design

The primary objective of this study was to ascertain public attitudes towards gene-based technologies/services with the potential for improving educational outcomes. To do this, we administered a cross-sectional, self-administered survey (see online supplemental material 1), which was adapted from a survey instrument designed by Meyer *et al* and used in a US population.^12^ Pilot testing was conducted with a panel comprising five academic staff to help adapt the survey for use in the Singaporean context and to obtain feedback on the time taken to complete the survey. This pilot panel was not assigned any group, and questions were reviewed across all three services. Substantial feedback gathered from the pilot panel was used to provide definitions for the terms ‘medical and non-medical traits’, ‘predictions’ and ‘SAT’ (Scholastic Assessment Test). These definitions were incorporated into the survey. Despite the panel’s suggestions to rephrase the questions, we maintained the original wording to ensure comparability with the findings from Meyer *et al*.

The final survey was developed, hosted on, and distributed via the Qualtrics XM survey software platform and comprised three sections.

#### Morality of IVF

This first section included brief information on what the IVF procedure would entail and a multiple-choice question on how IVF is perceived in terms of its morality. Based on the pilot panel’s feedback, we added in the information about the fate of the other unused embryos from IVF, which were ‘frozen and stored for future use, donated for research or disposed of’. Respondents were asked, in their opinion, whether IVF is morally acceptable, morally wrong or not a moral issue.

#### Moral acceptability of one of three services (PES, gene editing or SAT preparation courses)

This section contained a short description of the assigned service and two questions. The first question asked whether the service was perceived as morally acceptable, morally unacceptable or not a moral issue. This question elaborated that both PES and gene editing technologies are applied to increase the chances of a child having certain ‘medical and non-medical traits’, and that the service was safe. In the PES scenario, respondents were also told that this service uses a genetic test on each embryo to predict certain medical and non-medical traits and that these predictions are not 100% accurate. Medical traits refer to characteristics of an individual that are related to their health and potential medical conditions. Examples of medical traits can include blood type, genetic markers for specific diseases and allergies. On the other hand, non-medical traits are characteristics that are not directly related to a person’s health and potential medical conditions. Examples of non-medical traits are cognitive ability, creativity, height and appearance.

The second question asked how likely these respondents would use the service themselves, on a continuous scale from 0% (not likely to use) to 100% (very likely to use), if 10% of the population presently uses it to increase their child’s likelihood of attending a top-100 university. In both scenarios of PES and gene editing, respondents were asked to imagine that they are using IVF to have a baby. While in the SAT preparation courses scenario, they were told to imagine that they have a child who is studying in a junior college. The added context to this question included (1) the service was free and (2) the service would raise their chances of having such a child from 3% to 5%.

We used ‘A levels’ as an alternative term for ‘SAT’, considering that ‘A levels’ is the qualifying examination taken by Singaporeans for admission to local universities. In addition, we substituted the term ‘high school’ with ‘junior college’, which is the equivalent educational terminology in Singapore.

#### Socioeconomic demographics

This section consisted of questions on age, gender, ethnicity, highest educational level, self-rated health, religion and household income range.

### Data collection

In October 2023, 1438 Singaporeans were recruited from the Health Opinion Population Survey (HOPS) panel to complete this survey. HOPS is an online research panel run by the Centre for Biomedical Ethics for use in public health and ethics research (IRB ref: LH-18-011). The panel was primarily recruited from existing databases maintained at the Saw Swee Hock School of Public Health and through postal invitations mailed to households selected from a sampling frame of deidentified household addresses provided by the Singapore Department of Statistics (SingStat). The eligibility criteria for enrolling in this panel are Singapore citizen or permanent resident; age of 21 years and above; ability to read and understand English; frequent user of the internet; and personal email account holder.

As of October 2023, the panel had 2513 members. These members were randomly divided into three groups using the random generator command in Microsoft Excel and each group was sent email invitations to examine their views on either one of three services: PES, gene editing or SAT preparation courses. The SAT preparation courses were a non-genetic strategy that served as a baseline for the other two genetic services. The survey was live for 25 days, with reminder emails sent to non-responders on days 7 and 21. Respondents were each sent an unique link to complete the survey in the invitation email and could attempt the survey on their laptop, desktop computer or mobile phone device. Participants received an SGD$5 supermarket electronic voucher on completion. Unfinished surveys were not included in the analysis.

### Data analysis

Data analysis was conducted using SPSS (IBM V.19). Descriptive statistics (mean, SD and percentages) were used to present findings of demographic characteristics and attitudes towards IVF and the services. Bivariate analysis was conducted to examine the association between attitudes towards the services and each of the sociodemographic variables, using χ^2^ tests for categorical data, Student’s t-test for continuous data and one-way analysis of variance for more than two independent groups. To prepare our results to be comparable with Meyer *et al*’s, we condensed the age range categories into ‘all ages’ and ‘under 35 years’ and highest education level categories into ‘at least a bachelor’s degree’ and ‘no bachelor’s degree’. All tests were two sided, and a p<0.05 was considered statistically significant.

As the HOPS panel did not have sufficient respondents representing the minority ethnicities (ie, Malays and Indians), the survey data were weighted for ethnicity to ensure representativeness of the Singapore population. Analyses of this study were based on the weighted data set.

## Results

### Demographics

Survey invitations were sent via email to each member of the HOPS panel. A total of 1438 responses were received (57% response rate). 458 respondents were in the PES subgroup, 482 respondents in the CRISPR subgroup and 498 respondents in the SAT subgroup (see table 1 in online supplemental material 2). These characteristics (except ethnicity) were consistent with the numbers reported in the 2020 Singaporean Census. To ensure a more representative sample of the Singaporean population, study analyses were weighted through over-sampling of Malays and Indians.

#### Moral acceptability of IVF

When asked about their view on the moral acceptability of IVF, 688 (48%) of the respondents selected the option ‘morally acceptable’, 524 (36%) chose ‘not a moral issue’ while the remaining minority responded with ‘morally wrong’ (n=110, 8%) and ‘not sure’ (n=116, 8%).

#### Moral acceptability of each service and willingness to use

20% of the respondents viewed embryo selection for desirable medical and non-medical traits to be morally wrong (see table 2 in online supplemental material 2), compared with 31% who considered gene editing as morally wrong and 4% for SAT preparation courses. Mean willingness to use these services to increase their child’s chances of securing admission to a top-100 university was 57% for embryo selection, 48% for gene editing and 71% for SAT preparation courses. Less than half of the respondents (47%) indicated a likelihood of more than 50% to use gene editing, while more than half indicated such a likelihood for employing embryo selection (58%) and SAT preparation courses (78%).

#### Associations between respondent characteristics and (1) moral acceptability and (2) willingness to use the services

**Figure 1:**
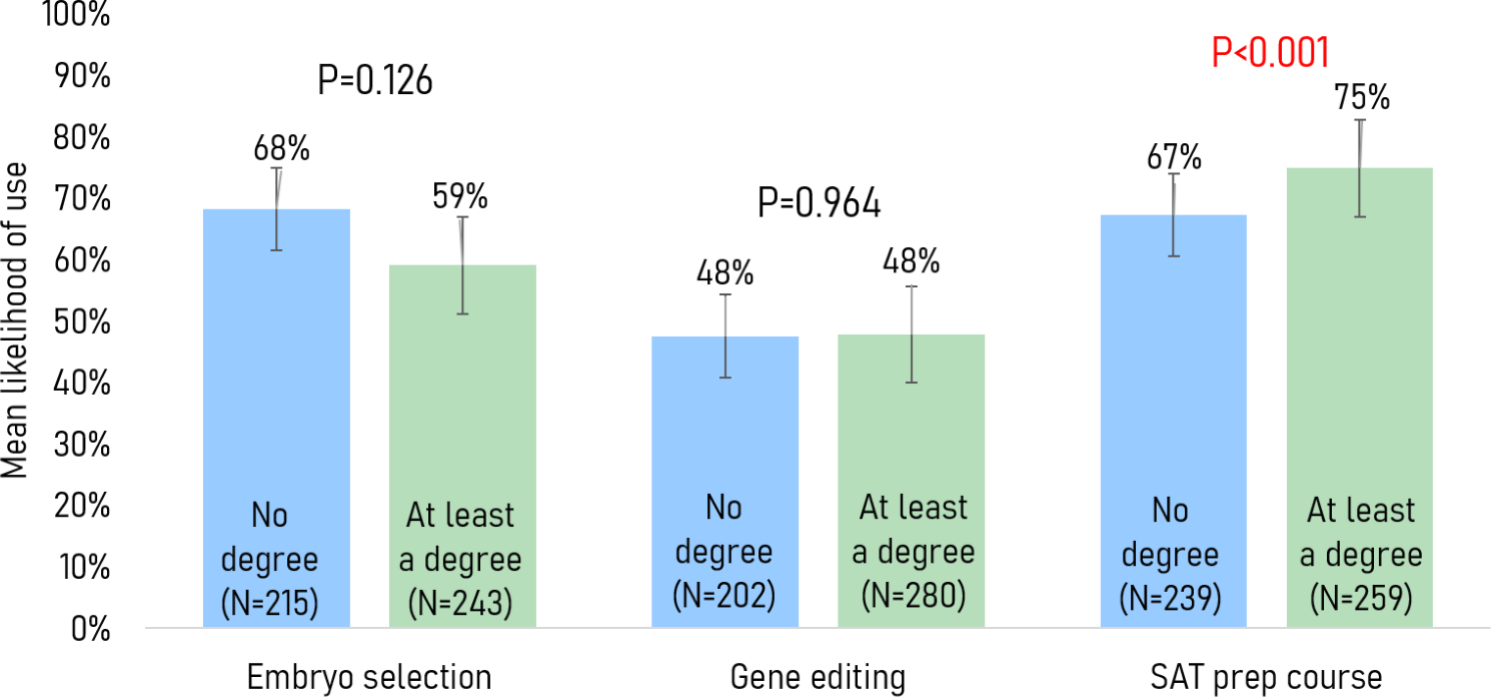
Mean willingness to use each service stratified by highest education level.

Highest education level: Respondents with at least a bachelor’s degree were more inclined to find SAT preparation courses morally acceptable or not a moral issue (94% vs 85%, p=0.002) (see table 3 in online supplemental material 2). No significant associations were found in mean willingness to use the genetic services (ie, embryo selection and gene editing) between respondents with at least a bachelor’s degree and those without. However, respondents with at least a bachelor’s degree had a greater likelihood on average of using SAT preparation courses to improve their child’s chances of enrolling in a top-100 university than those without a degree (75% vs 67%, p<0.001) (figure 1).

**Figure 2:**
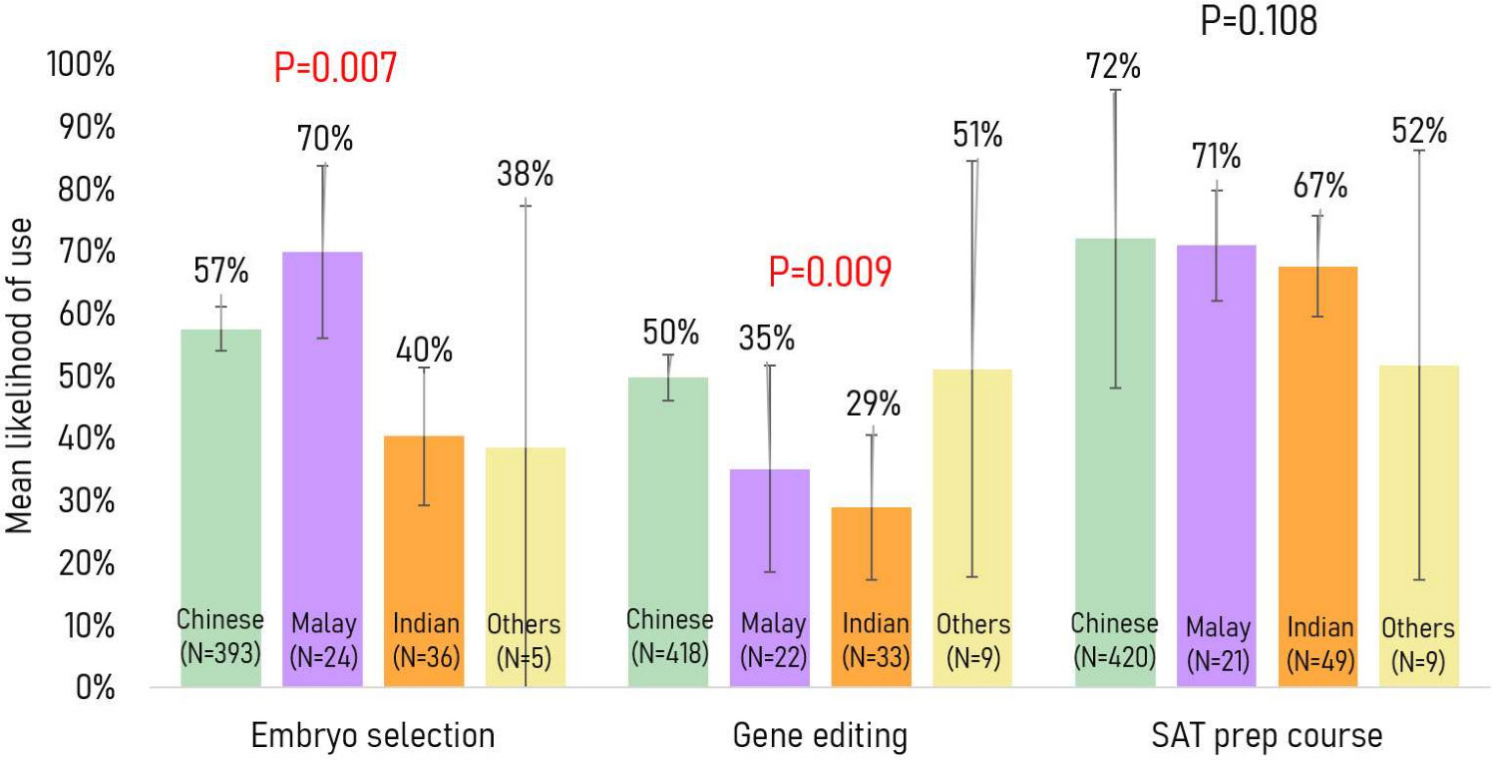
Mean willingness to use each service stratified by ethnicity.

Ethnicity: A significant association between ethnicity and mean willingness to use embryo selection was found, with higher willingness in Chinese (57%) and Malays (70%) compared with Indians (40%) (p=0.007) on average (figure 2). Chinese respondents (50%) were also, on average, more willing to use gene editing than Indians (29%) (p=0.009) (figure 2).

**Figure 3:**
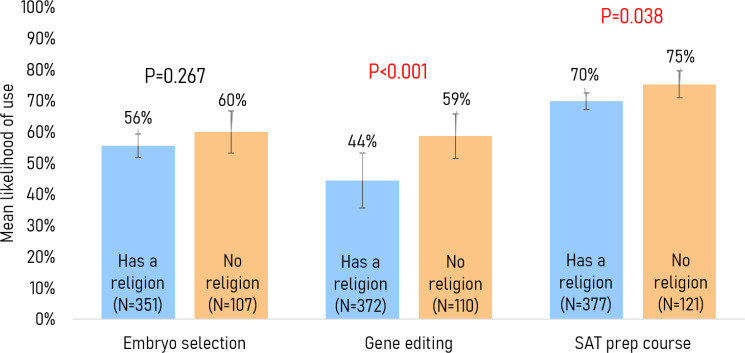
Mean willingness to use each service stratified by religion.

Religion: Compared with those without a religion, a smaller proportion of respondents with a religion viewed using gene editing to achieve desirable traits in their future child as morally acceptable or not a moral issue (49% vs 68%, p=0.004) (see table 4 in online supplemental material 2). Respondents with a religion were, on average, less inclined than those without any religious affiliations to use gene editing (44% vs 59%, p<0.001) and SAT preparation courses (70% vs 75%, p=0.038) (figure 3).

**Figure 4:**
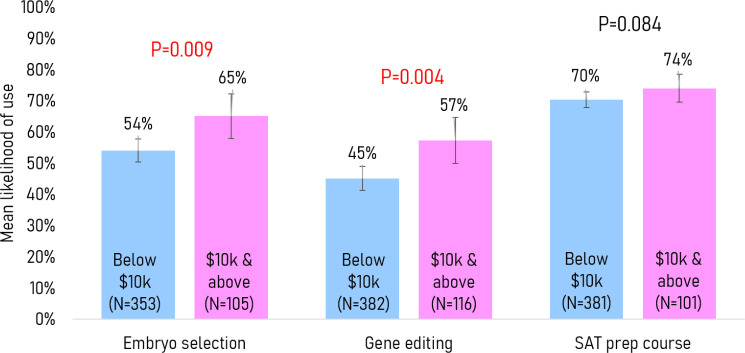
Mean willingness to use each service stratified by monthly household income.

Monthly household income: There was no significant association between the moral acceptability of the services and monthly household income. However, in the willingness to use question, a higher monthly household income of SGD$10 k and above was significantly associated with the greater likelihood of using both embryo selection (65% vs 54%, p=0.009) and gene editing (57% vs 45%, p=0.004), compared with a monthly household income of below SGD$10 k (figure 4).

Age and self-rated health: No significant associations were found between the attitudes towards the services and the demographic variables, age and self-rated health.

## Discussion

Compared with the equivalent US study, our results suggest that, among a Singaporean population, there appears to be greater moral acceptance of, and greater willingness to use PES, gene editing and SAT preparation courses to increase children’s chances of securing admission (see table 1 and table 2).^12^ ^iii^

**Table 1:** Comparing moral acceptance of genetic technologies between a Singaporean and US sample

Service	Morally acceptable		Morally wrong		Not a moral issue		Not sure	
	**Singapore**	**USA**	**Singapore**	**USA**	**Singapore**	**USA**	**Singapore**	**USA**
Embryo selection	40%	27%	20%	17%	28%	30%	13%	25%
Gene editing	32%	19%	31%	29%	22%	22%	15%	30%
SAT preparation courses	34%	27%	4%	7%	56%	49%	6%	16%

SAT, Scholastic Assessment Test.

**Table 2:** Comparing willingness to use genetic technologies between a Singaporean and US sample

Service	Willingness to use >50%		Mean willingness to use	
	**Singapore**	**USA**	**Singapore**	**USA**
Embryo selection	58%	38%	57%	43%
Gene editing	47%	28%	48%	34%
SAT preparation courses	78%	68%	71%	69%

SAT, Scholastic Assessment Test.

Such differences are likely to be attributable to differences in cultural values, particularly with respect to educational attainment. Singapore is a multiethnic, highly affluent society, which, like many East Asian countries, is influenced by Confucian values. This is reflected in Singapore’s hard-working culture, commitment to education, desire to attain better jobs and remuneration, and the fulfilment of cultural expectations to support multigenerational families.^18^ East Asian cultures are also renowned for their ‘tiger-parenting’, a type of parenting characterised by an achievement-orientated nature and tight scheduling of educational activities, which is especially prevalent among the Chinese population.^19^

Significantly, only 20% of Singaporeans thought PES for educational attainment was morally wrong, and only 31% thought gene editing was wrong, which suggests overall high acceptance among the population. Among the Singaporean sample, as was the case with the US sample, there appears to be greater acceptability of PES compared with gene editing. This may be because, unlike gene editing, PES does not require genetic manipulation or modification and is akin to picking a ‘winning ticket’ in a ‘genetic lottery’ based on the various possible combinations and permutations of genes that occur during fertilisation.^20^ The differences observed among ethnicities are also significant. Indeed, there was a higher percentage of Malays (70%) in support of PES compared with the Indian sample (40%). Furthermore, the Malay sample was significantly less supportive of gene editing (35%) compared with PES. Such differences may be attributable to the Islamic religion, given most Malays are Muslim.^20^ As Chin *et al* have observed previously, gene editing for the purpose of enhancement is forbidden in Islam, as it would imply altering Allah’s (Islamic God’s) creation. Since PES does not result in such alterations, man-made genetic alterations cannot be passed on and hence it may be more acceptable to that population.^20^ ^iv^ Moreover, the higher support for PES for cognitive enhancement among the Malay population may be influenced by sociocultural factors. Malays are a marginalised ethnic minority that are often outcompeted in higher education and jobs by other populations, and hence may see PES as a means to improve their children’s future opportunities.^21^ ^v^

Significantly, the desire for PES in and of itself as assessed by public attitudes surveys is not sufficient to introduce and implement PES on its own. While public views should not necessarily be determinative of policy implementation, they still have an important role to play with respect to shaping policy and could be used to inform a collective reflective equilibrium process.^22^ Current limitations with respect to the technology’s capability may suggest that such technology is not necessarily ready for clinical implementation at present,^1 4 6^ which may point to the need to further refine the technology prior to implementation. Indeed, while there are several limitations associated with PRS,^23^ of particular significance to the Singaporean population is the fact that many genome-wide association studies (GWAS) have been conducted on European populations.^24^ Given that PRS are less accurate for those of non-European ancestries, PRS may have limited generalisability to the Singaporean population. This provides further support for the need to diversify GWAS cohorts, so their results are more widely generalisable.

Similarly, concerns arise with the impact of pleiotropy (ie, where one or a set of genes affects multiple traits) and the influence of environment, and how this may impact the utility of PES or gene editing. Relevantly for this study, such concerns have previously been raised in the context of intelligence, given that it is known to be a highly polygenic trait.^25^ Indeed, studies have estimated that the heritability of intelligence is approximately 50%, and determining the contribution of an individual gene is challenging due to its minuscule effect.^26^ Additionally, accurately assessing intelligence as distinct from education or test training is currently challenging, although educational attainment is often used as a proxy for intelligence.^25^

While refinement in technology over time can address some of these limitations and enable clinical implementation, new technologies such as Brain Chip Technology^vi^ could outpace PES and genetic editing and render them obsolete.^27^ This resonates with previous criticisms that warn of today’s cognitively enhanced children becoming outdated due to further progress in genetic enhancement, such that they become ‘yesterday’s children’.^28^

There are also wider social and ethical implications that arise with the use of gene editing and PES. While it is not within the scope of this article to engage in a full analysis of these issues, some ethical concerns raised in this context include the potential for this to create social inequality and entrench unjust discrimination, impair parent–child relationships due to skewed expectations (and potentially disrupt family harmony) as well as expressivist, eugenic and justice concerns.^23 25^ Notably, such concerns are not universal. Indeed, some have also argued that we have an ethical duty to select for particular traits according to the principle of procreative beneficence advanced by Savulescu, which requires parents to select a child to have the best life or at least as good of life as others based on the information available,^29^ which has also been argued to apply in the gene editing context.^30^

Outside more normative concerns about whether these technologies should be introduced, consideration also needs to be given to appropriate regulatory approaches if such technologies were to be used for cognitive enhancement purposes as described in this survey, given PES screening for intelligence and gene editing for therapeutic (or clinical) purposes is currently not permitted in Singapore.^11 14^ In the context of PES, Chin *et al* have argued that Singapore’s heavy financial investment in education and pressure to succeed academically is likely to incentivise the use of such genetic technologies to improve the chances of academic success.^31^ Accordingly, the authors posited that this creates a potential for a particularly lucrative market for the widespread application of PES in Singapore and have called for regulatory safeguards to prevent the misuse of PES and safeguard the welfare and interests of the Singaporean population.^31^ Importantly, given that such technology is currently being used internationally, severe restrictions or a complete ban of the technology may encourage medical tourism. This, in turn, could exacerbate concerns surrounding social inequality, as without subsidies, only the most affluent would be able to access PES.^32^ In relation to Singaporean population, Chin *et al* have suggested a need for a rigorous regulatory framework to prevent abuse of the technology.^31^ The authors proposed that PES should only be used for disease traits and, hence, would not support PES for the use of educational attainment. They have also called for restrictions on accessing raw genomic data^vii^ and for rigorous and comprehensive genetic counselling.^31^

While our study demonstrated further support for the proposition that gene editing is generally less favourable than PES, our study did demonstrate that only a small proportion of participants perceived gene editing to be morally unacceptable. We note that in addition to the social and ethical issues with the use of gene editing for cognitive enhancement (some of which are discussed above), the technology is not error-free and does pose medical risks. Indeed, there is potential for such technology to result in errors such as deletions, insertions, rearrangements and chromosomal truncations.^33^ The risk of genetic mosaicism has also been identified previously.^34^ Many of these unintended effects will likely manifest later in life. Accordingly, if gene editing is to be introduced in the future, then efforts should be taken to refine the technology in addition to considering appropriate regulatory frameworks and necessary safeguards. However, given that gene editing is not being used clinically, and there have been calls by experts for a moratorium on its use,^35^ the use of gene editing for the purposes of educational attainment is less likely to be feasible in the short term.

### Limitations

One limitation of this study is that we relied on a sample from the HOPS panel, which is over-represented by young Chinese individuals and does not reflect the wider Singaporean population. Although data weighting has been applied, it would have been preferable to include more participants from minority groups, as well as non-English speaking cohorts. Future research should target such cohorts to ensure that they are more adequately represented.

Moreover, we did not assess participants’ awareness and understanding of cognitive enhancement including its potential benefits and limitations that were related to sociocultural aspects. Therefore, we were unable to assess the impact of knowledge on attitudes. Furthermore, while the survey did capture views about the acceptability of the various gene technologies, including for medical and non-medical traits, most of the focus was placed on educational attainment, so further research examining other traits is warranted. Finally, further qualitative research is required to understand the views of multiple stakeholder groups with respect to gene technologies including ascertaining any concerns they may have about their use, which should be used to inform future policies.

## Conclusion

This study demonstrated that there is some public support for the use of PES and gene editing among the Singaporean population, for the purposes of educational outcomes. Such support is comparatively higher than what was found in a survey carried out in the US context. While public attitudes should be used to inform policy-making, this is not sufficient to determine whether a particular genetic technology or service should be implemented or how it should be regulated. Further research and consideration of this issue in Singapore among a variety of stakeholders as well as other Asian populations is warranted.

## Supplementary material

10.1136/jme-2024-110490 online supplemental file 1

10.1136/jme-2024-110490 online supplemental file 2

## Data Availability

All data relevant to the study are included in the article or uploaded as supplementary information.

## Footnotes

PES and ESPS (Embryo Selection based on Polygenic Scores) are synonyms.

We note that Chinese scientist He Jiankui did use gene editing clinically to edit human embryos to make them HIV resistance prior to implantation. However, such a use was not lawful and remains restricted in most jurisdictions.

US figures contained in the tables obtained from Meyer *et al’s* (2023) Supplementary Material Table S2online supplemental material table S2.

Chin *et al* have discussed some more subtle and nuanced interpretation of Islamic Shariah law which may see PES conflict with Islamic principles, but there is no scope in this paper to discuss such nuances.

We thank the anonymous reviewer for pointing us to the literature which could inform hypotheses about the differences observed in the Malay population.

It has previously been proposed that Brain Chip Technology could be developed to improve IQ and then implanted into people to enhance their cognitive performance.

Chin *et al* developed this recommendation based on concerns raised about a potential slippery slope scenario where the technology can be exploited by patients to access their raw genomic data which they can then use for other purposes.
