# Supplementary material for: Singaporean attitudes to cognitive enhancement: a cross-sectional survey
Source: J Med Ethics. 2025 Feb 25;51(11):e110490. doi: 10.1136/jme-2024-110490 (PMC12573384; doi:10.1136/jme-2024-110490)
Supplement: online supplemental file 1 [file jme-51-11-s001.pdf]

Survey: Public attitudes towards using gene-based technologies/services with the potential for improving educational outcomes

Study aim

You are invited to complete this survey. This survey aims to examine public attitudes towards the use of different methods to increase the likelihood of future children attaining better examination scores and enrolling in top universities.

[next page] Practical instructions

This survey should take no more than 10 minutes to complete. Your participation is completely voluntary and you may stop at any time by closing your browser window. We will not keep any incomplete survey data.

[next page] Morality of in vitro fertilization (IVF) [All respondents to answer]

In vitro fertilization (IVF) is a way of having babies in which several eggs are removed from a woman's ovary, and each egg is combined with a man's sperm in a medical laboratory. Each fertilized egg is then grown in the laboratory for several days until it becomes a small group of cells called an embryo. One of these embryos is selected and placed in a woman's uterus to create a pregnancy. The other unused embryos are frozen and stored for future use, donated for research or disposed of.

Do you personally believe that using IVF is morally acceptable, morally wrong, or is it not a moral issue? Please choose the answer that corresponds most closely to your view.

1. Morally acceptable
2. Morally wrong
3. Not a moral issue
4. Not sure

[next page] ESPS Domain: Embryo Selection based on Polygenic Scores [Subgroup #1 to answer]

A new service is available to people using IVF who must choose one embryo to use to create a pregnancy. This service uses a genetic test on each embryo to predict certain medical and non-medical traits* each resulting child is likely to or may have. These predictions can then be used to choose which embryo to select. These predictions are probabilities, not certainties. This means that predictions from the genetic test are not 100% accurate. Instead, predictions give the selected embryo a higher chance of having more positive traits than other embryos that could be chosen.

*Medical traits refer to characteristics of an individual that are related to their health and potential medical conditions. Examples of medical traits can include blood type, genetic markers for specific diseases, and allergies. On the other hand, non-medical traits are characteristics that are not directly related to a person's health and potential medical conditions. Examples of non-medical traits are cognitive ability, creativity, height and appearance.

Do you personally believe that using this service is morally acceptable, morally wrong, or is it not a moral issue? Please choose the answer that corresponds most closely to your view.

1. Morally acceptable
2. Morally wrong
3. Not a moral issue
4. Not sure

[next page] ESPS domain and 10% public use [Subgroup #1 to answer]

Imagine that you are using IVF to have a baby.

You now have several embryos in a dish and must choose one embryo to create a pregnancy. A new service uses a genetic test on each embryo to predict which one is most likely to result in a child who attends a top-100 ranked university. Assume that you could raise your likelihood of having a child who attends a top-100 university to 5% (from 3%) by using this service. Imagine that this embryo selection service is safe and free, and on average it is used by 1 out of every 10 people currently having babies. How likely would you be to use this service?

Range: 0-100% (0%=Not likely to use, 100%=Very likely to use)

[next page] SAT Domain: SAT / A-levels Preparation Classes [Subgroup #2 to answer]

Many universities use Scholastic Assessment Test (SAT)*/ A-levels scores to decide which students to admit. SAT / A-levels preparation classes allow junior college students applying to universities to improve their results on the SAT /A-levels.

*The SAT is a standardised exam measuring students' ability in math, reading and writing. While the SAT is not required to apply to universities in Singapore, it is often required for application to universities overseas such as in the US and the UK.

Do you personally believe that using this service is morally acceptable, morally wrong, or is it not a moral issue? Please choose the answer that corresponds most closely to your view.

1. Morally acceptable
2. Morally wrong
3. Not a moral issue
4. Not sure

[next page] SAT domain and 10% public use [Subgroup #2 to answer]

Imagine that you have a child who is studying in a junior college. Many universities use SAT/A-levels scores to decide which students to admit. SAT/A-levels preparation classes allow junior college students applying to university to improve their results on the SAT/A-levels. Assume that you could raise the likelihood of having a child who attends a top-100 university to 5% (from 3%) by using this service. Imagine that this SAT/A-levels preparation service is free, and on average it is used by 1 out of every 10 people who currently have children who are attending a junior college. How likely would you be to use this service?

Range: 0-100% (0%=Not likely to use, 100%=Very likely to use)

[next page] CRISPR Domain: Gene Editing [Subgroup #3 to answer]

A new service could one day be offered to people using IVF to have a baby. With this service, before an IVF embryo is transferred to a woman's uterus to create a pregnancy, the embryo's genes are edited to increase the chance of the child having certain medical and non-medical traits*. Science has progressed and now gene editing is as safe as other medical treatments. With gene editing, segments of DNA are added, deleted, or corrected, and the child could pass down these changes to their own children.

*Medical traits refer to characteristics of an individual that are related to their health and potential medical conditions. Examples of medical traits can include blood type, genetic markers for specific diseases, and allergies. On the other hand, non-medical traits are characteristics that are not directly related to a person's health and potential medical conditions. Examples of non-medical traits are cognitive ability, creativity, height and appearance.

Do you personally believe that using this service is morally acceptable, morally wrong, or is it not a moral issue? Please choose the answer that corresponds most closely to your view.

1. Morally acceptable
2. Morally wrong
3. Not a moral issue
4. Not sure

[next page] CRISPR domain and 10% public use [Subgroup #3 to answer]

Imagine that you are using IVF to have a baby.

Suppose that a new service can edit the genes of an embryo to increase the chance that the child attends a top-100 ranked university. The child could pass down these gene edits to their own children. Assume that you could raise your likelihood of having a child who attends a top-100 university to 5% (from 3%) by using this service. Imagine that this gene editing service is safe and free, and on average it is used by 1 out of every 10 people currently having babies. How likely would you be to use this service?

Range: 0-100% (0%=Not likely to use, 100%=Very likely to use)

[next page] Demographics [All respondents to answer]

Age (in years): __________

Gender:

1. Male
2. Female

Race:

1. Chinese
2. Malay
3. Indian
4. Others (please specify): ____________

Religion:

1. Buddhism
2. Catholicism
3. Christianity
4. Hinduism
5. Islam
6. Taoism
7. Sikhism
8. No religion
9. Others (please specify): ________________

Highest education level:

1. No formal education
2. Pre-Primary
3. Primary
4. Secondary
5. Post-Secondary
6. Polytechnic
7. A Levels
8. Other Diploma
9. University- Bachelor’s Degree
10. University- Postgraduate Degree

Monthly household income:

1. No income
2. $2,999 and below
3. $3,000 to $5,999
4. $6,000 to $9,999
5. $10,000 to $14,999
6. $15,000 & above

How would you rate your health?

1. Excellent
2. Very good
3. Good
4. Fair
5. Poor
