# Supplementary material for: Singaporean attitudes to cognitive enhancement: a cross-sectional survey
Source: J Med Ethics. 2025 Feb 25;51(11):e110490. doi: 10.1136/jme-2024-110490 (PMC12573384; doi:10.1136/jme-2024-110490)
Supplement: online supplemental file 2 [file jme-51-11-s002.pdf]

Supplementary Material 2

Table 1. Respondent characteristics

Respondent's characteristic	No. of respondents (%)
Age group (years)	
21 - 30	147 (10%)
31 - 40	332 (23%)
41 - 50	326 (23%)
51 - 60	277 (19%)
61 - 70	269 (19%)
71 - 80	91 (6%)
81 - 90	4 (1%)
Gender	
Female	854 (59%)
Male	584 (41%)
Ethnicity	
Chinese	1231 (86%)
Malay	67 (5%)
Indian	118 (8%)
Others	22 (2%)
Highest education level	
No formal education	2 (1%)
Pre-Primary	2 (1%)
Primary	9 (1%)
Secondary	189 (13%)
Post-Secondary	78 (5%)
Diploma	316 (22%)
A Levels	60 (4%)
University - Bachelor's Degree	604 (42%)
University - Postgraduate Degree	178 (12%)
Religion	
Buddhism	367 (26%)
Christianity	400 (28%)
Catholicism	92 (6%)
Hinduism	65 (5%)
Islam	99 (7%)
Sikhism	6 (1%)
Taoism	65 (5%)
Others	6 (1%)
No religion	338 (24%)
Monthly household income	
No income	185 (13%)

$2,999 and below	207 (14%)
$3,000 to $5,999	394 (28%)
$6,000 to $9,999	324 (23%)
$10,000 to $14,999	182 (13%)
$15,000 & above	146 (10%)
Self -rated health	
Excellent	125 (9%)
Very good	430 (30%)
Good	679 (47%)
Fair	190 (13%)
Poor	14 (1%)

Table 2. Moral acceptability and willingness to use service

Service	Morally acceptable	Morally wrong	Not a moral issue	Not sure	Willingness to use > 50% (standard error)	Mean willingness to use (standard error)
Embryo selection (*N*=458)	182 (40%)	92 (20%)	126 (28%)	58 (13%)	266 (58%)	57% (1.69)
Gene editing (*N*=482)	152 (32%)	151 (31%)	105 (22%)	74 (15%)	226 (47%)	48% (1.76)
SAT preparation courses (*N*=498)	168 (34%)	20 (4%)	280 (56%)	30 (6%)	387 (78%)	71% (1.14)

Table 3: Moral acceptability of each service stratified by highest education level

Service	Highest education level	"Moral acceptable" or "not a moral issue"	"Morally wrong"	P-value
Embryo selection (N=458)	No bachelor's degree (N=215)	145 (67%)	37 (17%)	0.224
	At least a bachelor's degree (N=243)	163 (67%)	55 (23%)	
Gene editing (N=482)	No bachelor's degree (N=202)	113 (56%)	58 (29%)	0.502
	At least a bachelor's degree (N=280)	144 (51%)	93 (33%)	
SAT preparation courses (N=498)	No bachelor's degree (N=239)	204 (85%)	9 (4%)	0.002
	At least a bachelor's degree (N=259)	244 (94%)	11 (4%)	

Table 4. Moral acceptability of each service stratified by religion

Service	Religion	"Moral acceptable" or "not a moral issue"	"Morally wrong"	P-value
Embryo selection (N=458)	Has a religion (N=351)	235 (67%)	74 (21%)	0.161
	No religion (N=107)	73 (68%)	18 (17%)	
Gene editing (N=482)	Has a religion (N=372)	182 (49%)	130 (35%)	0.004
	No religion (N=110)	75 (68%)	21 (19%)	

SAT preparation courses (N=498)	Has a religion (N=377)	338 (90%)	16 (4%)	0.958
	No religion (N=121)	110 (91%)	4 (3%)	
